# Discrepancies between implicit and explicit self-concepts of intelligence: relations to modesty, narcissism, and achievement motivation

**DOI:** 10.3389/fpsyg.2014.00085

**Published:** 2014-02-10

**Authors:** Friederike X. R. Gerstenberg, Roland Imhoff, Rainer Banse, Manfred Schmitt

**Affiliations:** ^1^Department of Psychology, Technische Universität MünchenMunich, Germany; ^2^University of CologneCologne, Germany; ^3^University of BonnBonn, Germany; ^4^University of Koblenz-LandauLandau, Germany

**Keywords:** intelligence self-concept, implicit association test, intelligence, modesty, narcissism, achievement motivation

## Abstract

Previous research has shown that different configurations of the implicit self-concept of intelligence (iSCI) and the explicit self-concept of intelligence (eSCI) are consistently related to individuals’ performance on different intelligence tests (Dislich etal., 2012). The results indicated that any discrepant configuration between the iSCI and the eSCI impairs performance. In the present study, how correspondence between the iSCI and the eSCI is related to intelligence test performance as well as personality traits of modesty (low eSCI, high iSCI), narcissism (high eSCI, low iSCI), and achievement motivation was investigated. Furthermore, a moderated mediation analysis showed that the relation between the iSCI–eSCI configurations and intelligence test performance was mediated by achievement motivation for modest individuals.

## INTRODUCTION

Previous research by Dislich et al. (2012) has provided evidence that the interplay of the explicit self-concept of intelligence (eSCI) and the implicit self-concept of intelligence (iSCI) is systematically related to actual performance on well-established intelligence tests. High eSCI was generally related to higher performance, but this relation was moderated by the iSCI. Whenever the implicit association contradicted the eSCI, subsequent performance was impaired [a pattern, fully replicated more recently by Gerstenberg et al. (2013)]. More specifically, a *fragile* SCI (i.e., an explicit claim of being intelligent that was accompanied by a weak automatic association of the self with intelligence; high eSCI, low iSCI) was associated with reduced performance on intelligence tests compared to a *consistently high* SCI (high eSCI, high iSCI). Participants who self-reported not being very intelligent (low eSCI) performed generally worse on the IQ test, but this was specifically pronounced for those with an accompanying automatic association as intelligent (i.e., for *modest *SCI; low eSCI, high iSCI). The latter finding in particular invites speculation about why holding an iSCI as intelligent would impair test performance. In the present paper as a first goal we sought to explore the relation between SCI configurations and personality variables. As a second goal, we tested whether different levels of achievement motivation could explain the obtained performance pattern by Dislich et al. (2012).

To empirically test the validity of the descriptive labels used by Dislich et al. (2012), we examined the relation between the modest type in the four-category SCI framework and the trait of modesty. It was assumed that individuals with the combination of high iSCI and low eSCI would have positive internal self-views but would not express them. Thus, individuals with a modest SCI pattern were expected to score higher on an assessment of the trait modesty. Overall, we expected individuals with a low eSCI and high iSCI to be more modest than all other combination of SCI individuals (*Hypothesis 1*).

In contrast to this modest type, individuals with a fragile SCI overestimate rather than underestimate their intelligence. Their bold explicit claim of intelligence is not accompanied by an identical automatic association of the self with intelligence. Whereas Dislich et al. (2012) have relied on the term *fragile* to label this SCI configuration, others have used the term *narcissistic* to characterize a positive explicit self-evaluation that is accompanied by a negative automatic implicit self-evaluation (e.g., Bosson et al., 2003; Golec de Zavala et al., 2009). In line with this label and the mask-model of narcissism (Bosson et al., 2003), some authors have found that individuals with discrepant self-evaluations (low implicit, high explicit) did indeed score high on self-reported measures of narcissism (e.g., Golec de Zavala et al., 2009; but see Gregg and Sedikides, 2010). In other words, narcissists are thought to simultaneously hold positive conscious self-views and harbor significant self-doubts at less conscious levels. Thus, the iSCI may contradict the positive eSCI due to its less conscious nature or due to a different susceptibility to self-presentational concerns. Independent of the exact mechanism, the cited theories suggest the prediction that, in the domain of SCI as well, individuals with a *fragile* (or *narcissistic*) configuration will have the highest scores on a narcissism questionnaire (*Hypothesis 2*).

A second goal of the present study was to test the mediating role of achievement motivation to account for the effects shown by modest individuals. Across all three studies by Dislich et al. (2012), individuals with a modest SCI performed similarly to or more poorly than individuals with a consistently low SCI. This result is particularly intriguing given that a positive iSCI is commonly assumed to be derived from repeated exposure to experiences (DeHart et al., 2006) and feedback that characterize the self as intelligent. If this plausible assumption is true, it is all the more surprising that modest individuals perform poorly on performance-based assessments of intelligence, as the effect could not be attributed to actual performance potential. Importantly, potential does not automatically translate into performance but an individual needs to have sufficient motivation to display and realize one’s potential. Otherwise, underachievement will be the inevitable result. Thus, to better understand and possibly explain the results found by Dislich et al. (2012), we propose a mediating effect of low achievement motivation. For individuals with a modest SCI, being intelligent might not be of central importance, which might lead to downplaying their intellectual abilities (low eSCI). At the same time, their lack of interest in intellectually outperforming their peers should be reflected by a low achievement motivation.

The present study was designed to test the assumption that modest individuals perform worse on intelligence tests and have the lowest achievement motivation (*Hypothesis 3*). Furthermore, we postulated that achievement motivation would mediate the relation between SCI and performance scores on an intelligence test (*Hypothesis 4*).

## MATERIALS AND METHODS

### PARTICIPANTS AND PROCEDURE

A total of 84 students (60 women, 24 men) enrolled in different majors at two German universities, with a mean age of 22.56 (*SD* = 3.6) participated in this study. The study was conducted in the laboratory in group sessions of up to six individuals. Upon arrival, participants were seated at individual computer stations where they completed the measure of iSCI (SCI-IAT) followed by the questionnaire measure of eSCI. Participants then responded to the modesty scale, the narcissism questionnaire, and the achievement motivation scale. Finally, they were asked to complete the performance-based assessment of intelligence, a multiple-choice vocabulary test, and then they were fully debriefed and thanked.

### MEASURES

#### Implicit self-concept of intelligence

For the SCI-IAT, we used the same stimuli as proposed by Dislich et al. (2012). That is, for the target categories, we used the labels “me” versus “not me,” whereas for the attribute categories, we used “intelligent” versus “stupid.” For the target categories, we used the stimuli “me,” “my,” “mine,” “self,” “not me,” “you,” “yours,” “theirs,” and “it.” For the attribute categories we used the stimuli “intelligent,” “bright,” “clever,” “able,” “wise,” “stupid,” “dumb,” “foolish,” “silly,” and “dense.” We applied the standard IAT procedure (Greenwald et al., 1998) and calculated the IAT effects using the improved scoring algorithm proposed by Greenwald et al. (2003). We estimated the internal consistency of the SCI-IAT by computing two standardized difference scores for the odd trials in both critical blocks and the even trials in both critical blocks. These two *d*-scores were then used to estimate a Spearman–Brown-corrected split-half correlation.

#### Explicit self-concept of intelligence

An eight-item short version of the standardized inventory for measuring self-estimated intelligence (Rammstedt and Rammsayer, 2002) was used to assess participants’ eSCI. The items reflect seven primary mental abilities postulated by Thurstone (1938). An eighth item reflected general intelligence (e.g., Spearman, 1904). For each type of intelligence, a short description was provided (e.g., word fluency: “efficient and adequate expression of words”). Participants responded to the items by using a visual analog scale (scaled from 0 to 100) to indicate how well each type of intelligence characterized them.

#### Intelligence test

Intelligence was assessed using a multiple-choice vocabulary test (MWTA; Mehrfachwahl*–*Wortschatztest Form A; Lehrl et al., 1991). This test measures crystallized intelligence, which is the ability to use skill, knowledge, and experience and which relies on information from long-term memory. Its concurrent validity with other standard measures of crystallized intelligence has been provided by Lehrl et al. (1991). We decided to use the MWTA as it was also used in the studies by Dislich et al. (2012) and Gerstenberg et al. (2013). The MWTA consists of 37 items of increasing difficulty. Scores on the MWTA can range from 0 to 37 and are then recalculated into typical IQ scores with a mean of 100 and a *SD* of 15. For each trial, examinees were shown a list of five words (one real word and four non-words) and were asked to choose the real world from the list.

#### Modesty

Modesty was measured using a subscale of the German version of the NEO Personality Inventory-Revised [NEO PI-R; Costa and McCrae (1992); German version: Ostendorf and Angleitner (2004)]. Modesty is a facet of the major factor agreeableness. Modesty as measured by the NEO PI-R is the tendency to play down one’s own achievements and to be humble. The subscale consists of eight items that are rated on six-point scales.

#### Narcissism

Narcissism was measured using the German version of the Narcissistic Personality Inventory [NPI; Raskin and Hall (1979); German version: NPI 40; Schütz et al. (2004)]. This instrument measures narcissism as a personality trait relating to a love of self and self-absorption. It is typically used to study sub-clinical narcissism. The NPI 40 contains 40 true-false statements (e.g., “I will be a success.” vs. “I am not too concerned about success.”).

#### Achievement motivation

Achievement motivation was measured using the German version of the Achievement Motives Scale [AMS; Gjesme and Nygard (1970); German version: Lang and Fries (2006)]. The AMS contains 10 items that are rated on four-point scales.

## RESULTS

All indicators were scaled such that higher scores indicated higher levels of the respective construct. Descriptive statistics, internal consistencies (Cronbach’s alpha), and intercorrelations for all measures are presented in **Table 1**. Similar to the results of Dislich et al. (2012) and Gerstenberg et al. (2013), the SCI-IAT and the eSCI were not found to be significantly correlated.

**Table 1:** Descriptive statistics and correlations for all variables.

	(1)	(2)	(3)	(4)	(6)	(7)
(1) iSCI (IAT, D measure)	–					
(2) eSCI	0.39**	–				
(3) Crystallized intelligence (MWTA)	0.17	0.49**	–			
(4) Modesty	0.29*	-0.11	-0.10	–		
(5) Narcissism	-0.14	0.33**	0.07	-0.11	–	
(6) Achievement motivation	-0.14	0.44**	0.45**	-0.20	0.03	–
#	NA	8	34	8	40	10
*α*	0.72	0.89	0.81	0.87	0.88	0.82
*M*	0.63	59.46	103.01	13.26	17.78	22.51
*SD*	0.34	11.91	9.09	2.19	3.19	3.78
Min	-0.23	34.33	85	7	11	9
Max	1.29	87.67	128	18	28	31

*N* = 84; *#* = number of items; *α* = internal consistency (Cronbach’s alpha); iSCI = implicit self-concept of intelligence; eSCI = explicit self-concept of intelligence; MWTA = multiple-choice vocabulary test. *p < 0.05, **p < 0.01.

### MULTIPLE REGRESSION ANALYSES

To determine whether the iSCI and eSCI were related to scores on the intelligence test, the modesty scale, the narcissism questionnaire, and the achievement motivation scale, multiple regression analyses were used. The dependent variables were regressed onto centered eSCI scores, centered iSCI scores, and their interaction term. Results of the regression analyses are displayed in **Table 2**. The regression analyses were followed by simple slope tests.

**Table 2:** Regression analyses predicting intelligence test performance, modesty, narcissism, and achievement motivation.

	*R*^2^	β
Intelligence test	0.29**	
iSCI		0.15
eSCI		0.48**
iSCI × eSCI		0.23*
Modesty scale	0.08*	
iSCI		0.23*
eSCI		-0.12
iSCI × eSCI		-0.21*
Narcissism scale	0.18**	
iSCI		-0.16
eSCI		0.34**
iSCI × eSCI		-0.27**
Achievement motivation	0.24**	
iSCI		-0.15
eSCI		0.44**
iSCI × eSCI		0.23*

iSCI = implicit self-concept of intelligence; eSCI = explicit self-concept of intelligence. *p < 0.05, **p < 0.01.

#### Intelligence test

**FIGURE 1:**
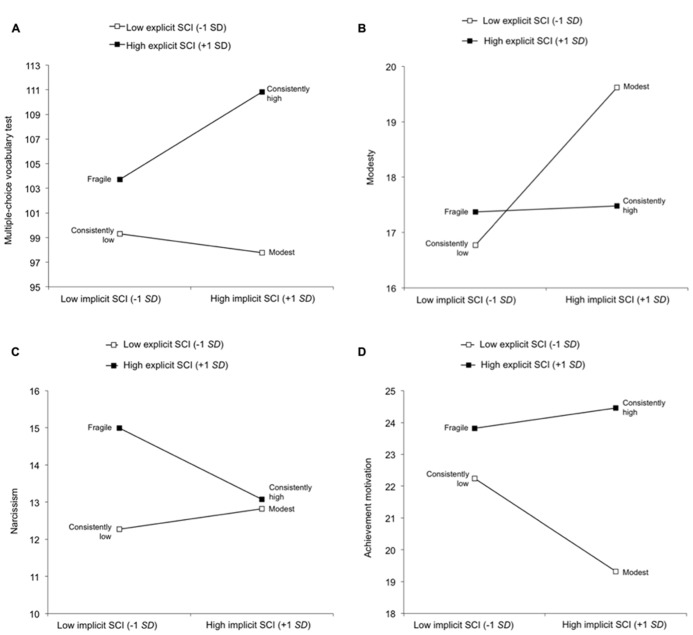
**(A)** Predicted values for the multiple-choice vocabulary test as a function of the explicit self-concept of intelligence and implicit self-concept of intelligence. **(B)****Predicted values for the modesty scale as a function of the explicit self-concept of intelligence and implicit self-concept of intelligence. **(C)****Predicted values for the narcissism scale as a function of the explicit self-concept of intelligence and implicit self-concept of intelligence. **(D)****Predicted values for the achievement motivation scale as a function of the explicit self-concept of intelligence and implicit self-concept of intelligence. SCI = self-concept of intelligence; SD = standard deviation.

Individuals with a high eSCI performed better than individuals with a low eSCI (see **Table 2**). The examination of simple slopes replicated the pattern of results found by Dislich et al. (2012), Studies 1–3 (**Figure 1A**). That is, individuals with a high eSCI (+1 SD) showed a significant positive relation between the iSCI and performance on the intelligence assessment, *B* = 3.55, *t*(81) = 7.26, *p* < 0.01. Individuals high on both types of SCI achieved significantly higher scores on the intelligence test than participants with a fragile SCI. The relation between the iSCI and scores on the intelligence test among individuals with a low eSCI (-1 SD) was also significant, indicating that modest individuals performed worse than persons with a consistently low SCI, *B* = -0.77, *t*(81) = 1.96, *p* < 0.05, supporting *Hypothesis 3*.

#### Modesty scale

Concerning the main effect of the iSCI (see **Table 2**), individuals with a low iSCI showed higher scores on the modesty scale than individuals with a high iSCI. **Figure 1B** shows a significant positive relation between modesty scores and the iSCI for individuals with a low eSCI, *B* = 1.42, *t*(81) = 3.61, *p* < 0.01, also supporting *Hypothesis 1*, which stated that individuals with a combination of a low eSCI and high iSCI are more modest than individuals with a congruently low SCI.

#### Narcissism scale

In general, individuals with a high eSCI scored higher on the narcissism scale than did individuals with a low eSCI (see **Table 2**). As can be seen in **Figure 1C**, there was a significant negative relation between NPI scores and the iSCI for individuals with a high eSCI, *B* = -0.96, *t*(81) = 1.96, *p* < 0.05. Thus, consistent with *Hypothesis 3*, individuals with a high eSCI and low iSCI scored higher on the narcissism questionnaire than individuals with a consistently high SCI.

#### Achievement motivation

The main effect of the eSCI indicated that individuals with a high eSCI achieved higher scores on achievement motivation than individuals with a low eSCI (see **Table 2**). The direction of the predicted interaction on the achievement motivation scale was positive, indicating that as the eSCI increased, the relation between a high iSCI and achievement motivation became stronger (see **Figure 1D**). Testing simple slopes above and below the mean of the eSCI, no significant positive relation was found between achievement motivation and the iSCI for individuals with a high eSCI, *B* = 0.32, *t*(81) = 0.65, *p* = 0.52. This finding supports *Hypothesis 3*, which stated that individuals with a fragile SCI would not differ in achievement motivation from individuals with a consistently high SCI. Furthermore, a significant negative relation was found between achievement motivation and the iSCI for individuals with a low iSCI, *B* = -1.46, *t*(81) = 3.71, *p* < 0.01. This relation is in line with *Hypothesis 3*, which predicted that individuals with a modest SCI would have lower achievement motivation than individuals with a consistently low SCI.

### MODERATED MEDIATION ANALYSIS

To test *Hypothesis 4*, which stated that achievement motivation would mediate the interaction effects of the iSCI and eSCI on intelligence test performance, additional analyses were performed. As previous analyses had already found a correlation between achievement motivation and intelligence test performance, and an effect from the two-way interaction of the iSCI and eSCI on the dependent variable as well as on the mediator, mediation would be demonstrated if simultaneously regressing the dependent variable on the predictors and the mediator resulted in a significant main effect of the mediator on the dependent variable and a reduced or zero effect on the two-way interaction. Intelligence test performance was regressed on the iSCI, eSCI, their interaction terms, and achievement motivation. The analysis, *R*^2^**= 0.34, revealed a significant main effect of achievement motivation, *β* = 0.29, *t*(80) = 2.77, *p *< 0.01, a significant main effect of the iSCI, *β* = 0.19, *t*(80) = 2.18, *p *< 0.05, and a significant main effect of the eSCI, *β* = 0.35, *t*(80) = 3.54, *p *< 0.01. In support of the assumed mediation, the two-way interaction between the iSCI and eSCI was no longer significant, *β* = 0.16, *t*(81) = 2.77, *p *= 0.08.

As we were specifically interested to test which configurations of the iSCI and eSCI produced indirect effects on intelligence test performance, we conducted bootstrapping analyses at specific values (*M* and *M* ± 1 *SD*) of the moderators (see Preacher and Hayes, 2004, 2008). For the resulting nine configurations, significance tests were conducted to test the hypothesis that the conditional indirect effect equals zero. Results revealed that of all possible combinations of the iSCI and eSCI, there was an indirect effect only for the modest combination (iSCI: +1 *SD*; eSCI: -1 *SD*), indicated by the fact that zero was outside the 95% confidence interval. Specifically, the region of significance had its lower bound at -1.122 and its upper bound at -0.086 with a mean conditional indirect effect of *β* = -0.47, *p* < 0.05. No other conditional indirect effect was significant.

## DISCUSSION

In the present study, the consistency of the iSCI and eSCI predicted the outcome of performance-based assessments of intelligence. Individuals with a fragile SCI performed worse on an intelligence test than individuals with a consistently high SCI, and individuals with a modest SCI performed worse than individuals with a consistently low SCI.

The hypotheses (*Hypotheses 1 *and* 2*) regarding the relation between the SCI and the personality traits of modesty and narcissism were also supported. Individuals with a high eSCI and low iSCI were expected to, and did, score higher on the narcissism scale. Individuals with a low eSCI and high iSCI were expected to, and did, score higher on the modesty scale. Somewhat surprisingly, the expected negative relation between modesty and narcissism was not significant. Although it is beyond the focus of the present paper, this finding calls for some attention. While the lack of a strong negative relation between narcissism and modesty may lead skeptics to doubt the validity of the scales, we would argue otherwise. As narcissists are highly motivated to present a favorable image of themselves, they may overestimate not only their performance but also the degree to which they hold desirable social attributes. If some narcissists choose to present themselves as grandiosely modest, this may undermine the theoretically expected negative correlation between the two. Future research may explore this finding and make use of techniques like the bogus pipeline to elucidate the relation.

Consistent with *Hypothesis 3*, achievement motivation was significantly predicted by the SCI. The obtained effects supported the hypothesis that individuals with a modest SCI have lower achievement motivation scores. Furthermore, the results supported the prediction that the relation between SCI and performance on the intelligence test would be mediated by achievement motivation for modest individuals (*Hypothesis 4*).

In line with the assumption that the iSCI reflects internalized experiences and feedback regarding intellectual abilities, narcissists’ impaired performance may also be a symptom of their intellectual abilities. The experience of repeated underperformance may lead to an implicit association of the self with low intelligence, an association that might be contradicted by strong claims of intelligence. Considering this idea that a low iSCI is the result of previous negative feedback, individuals with a narcissistic SCI performed surprisingly well compared to modest individuals on the intelligence test. It may be the case that narcissists’ explicit claim of being intelligent is indicative of an achievement-motivated coping mechanism in which narcissists counter their feelings of failure by working harder on a task. This assumption was somewhat supported by the fact that narcissists indeed showed a (at least descriptively) higher degree of achievement motivation than either consistently low or modest participants.

In the present study, a relation between modesty and lack of achievement motivation was found. These results can be explained by a mediating effect of achievement motivation, which can account for the relation between implicit–explicit SCI consistency and performance on intelligence tests. Thus, achievement motivation leads to increased performance on the intelligence test as a function of the underlying SCI structure. A similar effect has been obtained in the stereotype threat domain. Women’s mathematical performance was influenced by a reduced motivation to improve (Fogliati and Bussey, 2013). Obviously, as is true for any correlational study, any interpretation of causality warrants sufficient caution. Clearly, it is also conceivable that a low self-reported achievement motivation in modest participants is a defensive reaction to the anticipated bad performance in a sense of self-handicapping (“I only performed bad because I didn’t want to excel”). Although we have no data on which causal chain is accurate, we believe that the second suggestion has some greater problems. First, achievement motivation was measured before the IQ test and participants were not informed about a following IQ test. Thus, modest participants must have anticipated a performance test and negative performance and defensively self-handicapped prophylactically. Second, it is unclear why consistently low SCI would not lead to a similar reaction or, more specifically, why a high iSCI would lead to particularly strong defensive reactions. Third, research on the impact of negative feedback shows an exactly opposite pattern as individual with a modest SCI react with a boost in achievement motivation (labeled achievement-related reactance) to the information that they did not do well on an IQ test (Gerstenberg et al., 2013). Thus, although no claims of causality can be based on the current cross-sectional data, we believe that the proposed causal order stands out as the more plausible one.

The results of the present research also yielded important insights into the underlying motivational nature of the consistency of the iSCI and eSCI and its effects on intelligence test performance. The mediation results for achievement motivation allowed for a more nuanced understanding of the underlying mechanisms that drive the relation between implicit–explicit consistency than the mere identification of direct relations with outcome variables. The performance pattern on the intelligence test and the pattern of achievement motivation were very similar. Individuals with a modest SCI achieved the lowest intelligence test performance and achievement motivation scores.

In summary, the present work contributes to our understanding of the relation between self-concept consistency and performance on intelligence tests by demonstrating that the association of discrepant and consistent self-associations with performance differs systematically as a function of achievement motivation.

## Conflict of Interest Statement

The authors declare that the research was conducted in the absence of any commercial or financial relationships that could be construed as a potential conflict of interest.
